# Effectiveness of *Dunaliella salina* Extracts against *Bacillus subtilis* and Bacterial Plant Pathogens

**DOI:** 10.3390/pathogens9080613

**Published:** 2020-07-28

**Authors:** Alfredo Ambrico, Mario Trupo, Rosaria Magarelli, Roberto Balducchi, Angelo Ferraro, Evangelos Hristoforou, Tiziana Marino, Dino Musmarra, Patrizia Casella, Antonio Molino

**Affiliations:** 1 Energy and Sustainable Economic Development Department of Sustainability, ENEA Italian National Agency for New Technologies, R.C. Trisaia S.S. 106 Jonica, 75026 Rotondella, Italy; alfredo.ambrico@enea.it (A.A.); mario.trupo@enea.it (M.T.); rosaria.magarelli@enea.it (R.M.); roberto.balducchi@enea.it (R.B.); 2 School of Electrical and Computer Engineering, National Technical University of Athens, Zografou Campus, 9, IroonPolytechnioustr, 15780 Athens, Greece; ferraro@eie.gr (A.F.); hristoforou@ece.ntua.gr (E.H.); 3 Department of Civil and Building Engineering, Design and Environment, Università degli Studi della Campania “L.Vanvitelli”, Real Casa dell’Annunziata, Via Roma 9, 81031 Aversa, Italy; tiziana.marino@unicampania.it (T.M.); dino.musmarra@unicampania.it (D.M.); 4 Energy and Sustainable Economic Development Department of Sustainability, ENEA Italian National Agency for New Technologies, R.C. Portici, Piazzale Enrico Fermi 1, 80055 Portici, Italy; patrizia.casella@enea.it

**Keywords:** microalgae, antibacterial activity, algal bioactive compounds, *D. salina*, microalgae extracts, β-carotene, bacterial speck spot disease

## Abstract

Several bacteria pathogens are responsible for plant diseases causing significant economic losses. The antibacterial activity of *Dunaliella salina* microalgae extracts were investigated in vitro and in vivo. First, biomass composition was chemically characterized and subjected to extraction using polar/non-polar solvents. The highest extraction yield was obtained using chloroform:methanol (1:1 *v*/*v*) equal to 170 mg g^−1^ followed by ethanol (88 mg g^−1^) and hexane (61 mg g^−1^). In vitro examination of hexane extracts of *Dunaliella salina* demonstrated antibacterial activity against all tested bacteria. The hexane extract showed the highest amount of β-carotene with respect to the others, so it was selected for subsequent analyses. In vivo studies were also carried out using hexane extracts of *D. salina* against *Pseudomonas syringae* pv. *tomato* and *Pectobacterium carotovorum* subsp. *carotovorum* on young tomato plants and fruits of tomato and zucchini, respectively. The treated young tomato plants exhibited a reduction of 65.7% incidence and 77.0% severity of bacterial speck spot disease. Similarly, a reduction of soft rot symptoms was observed in treated tomato and zucchini fruits with a disease incidence of 5.3% and 12.6% with respect to 90.6% and 100%, respectively, for the positive control.

## 1. Introduction

Bacteria can have important functional roles in agriculture, for example, in interactions with soil, roots, and microorganisms, they can bring beneficial effects for the health and growth of plants; but, on the other hand, pathogenic bacteria can cause serious plant diseases.

Several Gram-negative and Gram-positive bacterial pathogens are responsible for plant diseases causing significant economic losses in crop production [1,2]. The pathogenic bacteria can be spread several ways including by rain, wind, birds, or insects [3,4]. In addition, a pathogen’s incidence may be favored by the propagation of plants with bacteria-infected material or by pruning infected trees [5]. Bacterial infections occur at any time during pre- and post-harvest processes reducing the quantity and quality of fruits and vegetables [6,7]. Gram-negative bacteria, such as *Pseudomonas syringae* and *Pectobacterium carotovorum*, are important bacteria in scientific and economic terms and can cause very substantial production losses and improper storage [8,9].

These pathogens typically enter the host through wound sites or natural openings, such as lenticels, and remain latent in intracellular spaces and vascular tissue [10,11,12]. Some environmental factors, such as temperature, low oxygen concentration, and free water, can affect disease progress. During favorable environmental conditions, bacterial pathogens produce a cellulolytic multi-enzyme complex that cause plant cell lysis and tissue collapse [13]. Nowadays, recent European restrictions on the use of chemicals and antibiotics for the management of bacterial diseases in agriculture have been enacted [14]. Copper-based treatments, despite being non-environmentally friendly and having a negative impact on microorganisms in soil, phyllosphere, and rhizosphere, are principally adopted to control bacterial plant diseases [15,16], while host resistance and appropriate agronomic practices, such as seeds certification, irrigation, and fertilization, are the principal strategies for integrated control of bacterial diseases in fields. During post-harvest, the more effective solutions to prevent bacterial diseases are proper storage conditions and good handling practices such as cleaning and disinfestations of equipment [17]. Therefore, alternative solutions are becoming essential to preserve the freshness and quality of food products. Natural compounds derived from microalgae can be exploited for these objectives due to the fact of their promising antibacterial properties [18,19].

Microalgae are aquatic photosynthetic organisms, converting CO_2_ and light into valuable, energy-rich organic compounds. In aquatic environments, prokaryotic and eukaryotic microalgae are annually responsible for about 50% of carbon fixation [20]. Microalgae are cultivated in photo-bioreactors or open systems for different final applications and purposes such as human consumption. Microalgae are indeed a source of high value healthy compounds like carotenoids and polyunsaturated fatty acids [21,22] whose systematic examination began in the 1950s [23,24]. At the same time, early in vivo observations about the pharmaceutical properties of microalgae strains were conducted in 1970 at the Roche Research Institute of Marine Pharmacology in Australia [25]. Recently, several studies have focused on evaluating in vitro and in vivo antimicrobial proprieties of algae [22,25] and microalgae extracts [19], such as *Botryococcus braunii* [21] and *Scenedesmus* sp. [18], that can produce different compound-shaving antibiotic, antioxidant, and antimicrobial properties that can potentially damage pathogenic cells. Therefore, novel natural antimicrobial molecules derived from microalgae can be investigated for crop protection [26,27]. From these natural antimicrobial substances, carotenoids are showing very promising results [28,29].

According to our knowledge, *Dunaliella salina* (a unicellular, biflagellate, naked green alga) represents an important source of β-carotene which has been investigated for its antimicrobial proprieties [30,31,32,33]. The aim of the present study was to evaluate the antibacterial proprieties of *D. salina* extracts.

The antibacterial effects of *D. salina* extracts were evaluated via in vitro and in vivo experimental trials. The antibacterial activity of *D. salina* extracts were compared with that of the β-carotene chemical standard.

## 2. Results

### 2.1. Extraction Yield and Characterization Pre- and Post-Extraction

*Dunaliella salina* chemical composition was characterized in terms of humidity, ash that amounted to 6.63% (*w*/*w* on wet sample), and 48.74% (*w*/*w* on wet sample) as reported in Table 1. Carbohydrates were the main constituent with a percentage equal to 25.31% (*w*/*w* on dry basis). Proteins and total dietary fiber (TDFs) were equal to 10.03% (*w*/*w* on dry basis) and 8.97% (*w*/*w* on dry basis). Carotenoids amounted to 3.46% and lipids were equal to 3.49% (*w*/*w* on dry basis). Fatty acids methyl esters (FAMEs) composition was investigated and saturated fatty acids (SFAs) and polyunsaturated fatty acids (PUFAs) were the most abundant: 1532.68 mg 100 g^−1^ on a dry basis and 1055.97 mg 100 g^−1^ on a dry basis, respectively (Table 1).

**Figure 1:**
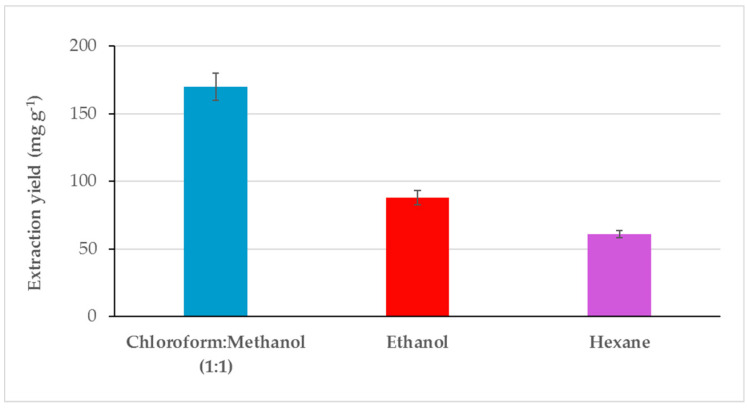
Extraction yield obtained by using solvents with different polarity.

*Dunaliella salina* dry biomass was extracted using solvents with different polarity. Ethanol was chosen since this is a Generally Recognized As Safe (GRAS) solvent, directly usable in food and pharmaceutical industries and allowed to obtain an extraction yield comparatively lower than that of chloroform:methanol (1:1) mixture but higher than that obtained with hexane (Figure 1). The solvent polarity is an important factor in estimating affinity of compounds with similar polarity. The extraction yield of *D. salina* biomass using chloroform:methanol (170 mg g^−1^) was two- and three-fold higher in respect to those obtained with ethanol (88 mg g^−1^) and hexane (61 mg g^−1^) (Figure 1).

For each extraction test, the chemical composition of *D. salina* extracts were evaluated as shown in Table 2. Hexane extract *D. salina* showed the highest content of carotenoids (36.6%) in respect to ethanol (12.8%) and chloroform:methanol (1:1 *v*/*v*) (16.4%) extracts. The ß-carotene amount was 98% of the total carotenoids in the hexane extract. The chloroform:methanol (1:1) extract of *D. salina* contained the highest protein content (60.6%), while the ethanol extract contained the highest lipids content (18.8%).

### 2.2. In Vitro Antimicrobial Activity of Different Extracts of Microalgae

#### 2.2.1. Disc Diffusion Method

The antibacterial properties of *D. salina* extracts were screened by the disc diffusion method (Table 3). All extracts of *D. salina* displayed an antibacterial activity against all tested bacteria. Specifically, hexane extracts of *D. salina* at the concentration of 97.0 mg mL^−1^ showed a higher inhibition zone equal to 20 mm against *B. subtilis* (BS), while the inhibition zones against *P. carotovorum* subsp. *carotovorum* (PCC) and *P. syringae* pv. *tomato* (PST) amounted to 9 and 12 mm, respectively. Ethanolic extracts at the concentration of 214.0 mg mL^−1^ showed inhibition zones of 21 mm against the BS strain, 11 mm against the PCC strain, and 9 mm against the PST strain. The chloroform–methanol extract was used at the concentration of 350 mg mL^−1^, and the obtained inhibition zone was 10, 8, and 13 mm against *P. carotovorum* subsp. *carotovorum*, *P. syringae* pv. *tomato*, and *B. subtilis*, respectively. The ciprofloxacin antibiotic was tested at 0.15 mg mL^−1^ showing an inhibition zone 32 mm against the BS strain, 24 mm against the PST strain, and 20 mm against the PCC strain.

#### 2.2.2. Minimum Inhibitory Concentration

For the determination of the minimum inhibitory concentration (MIC) values, *D. salina* extracts were subjected to the broth dilution method. After incubation time, when in control tubes, the microbial biomass was well grown, the inhibitory activity of *D. salina* extracts were evaluated. The results in Table 4 showed that for hexane extracts of *D. salina*, the MIC was achieved at 3.0 mg mL^−1^ and only against *B. subtilis* at 0.3 mg mL^−1^. Chloroform:methanol extracts were active only against *B. subtilis* with an MIC value of 3.0 mg mL^−1^, while ethanolic extracts of *D. salina* inhibited the growth of *P. syringae* and *B. subtilis* at a concentration of 3.0 mg mL^−1^.

### 2.3. Effect of D. salina Extracts on Disease Development in In Vivo Conditions

#### 2.3.1. Application of *D. salina* Extracts to Control Bacterial Speck Spot Caused by *P. syringae*

**Figure 2:**
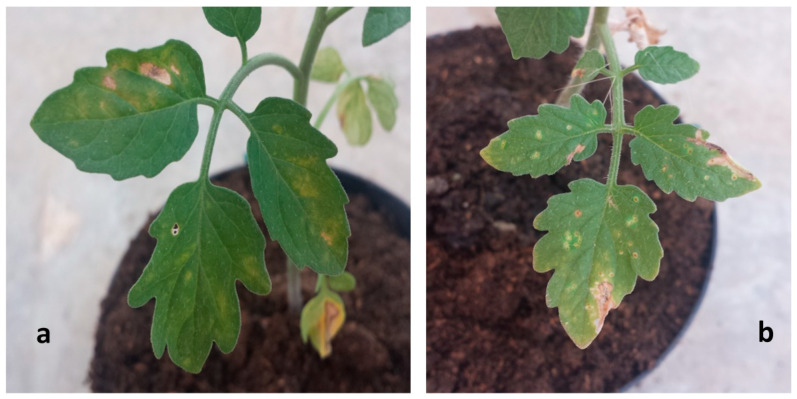
Symptoms of bacterial speck disease on inoculated tomato leaves after 10 (**a**) and 15 (**b**) days post-inoculation.

**Figure 3:**
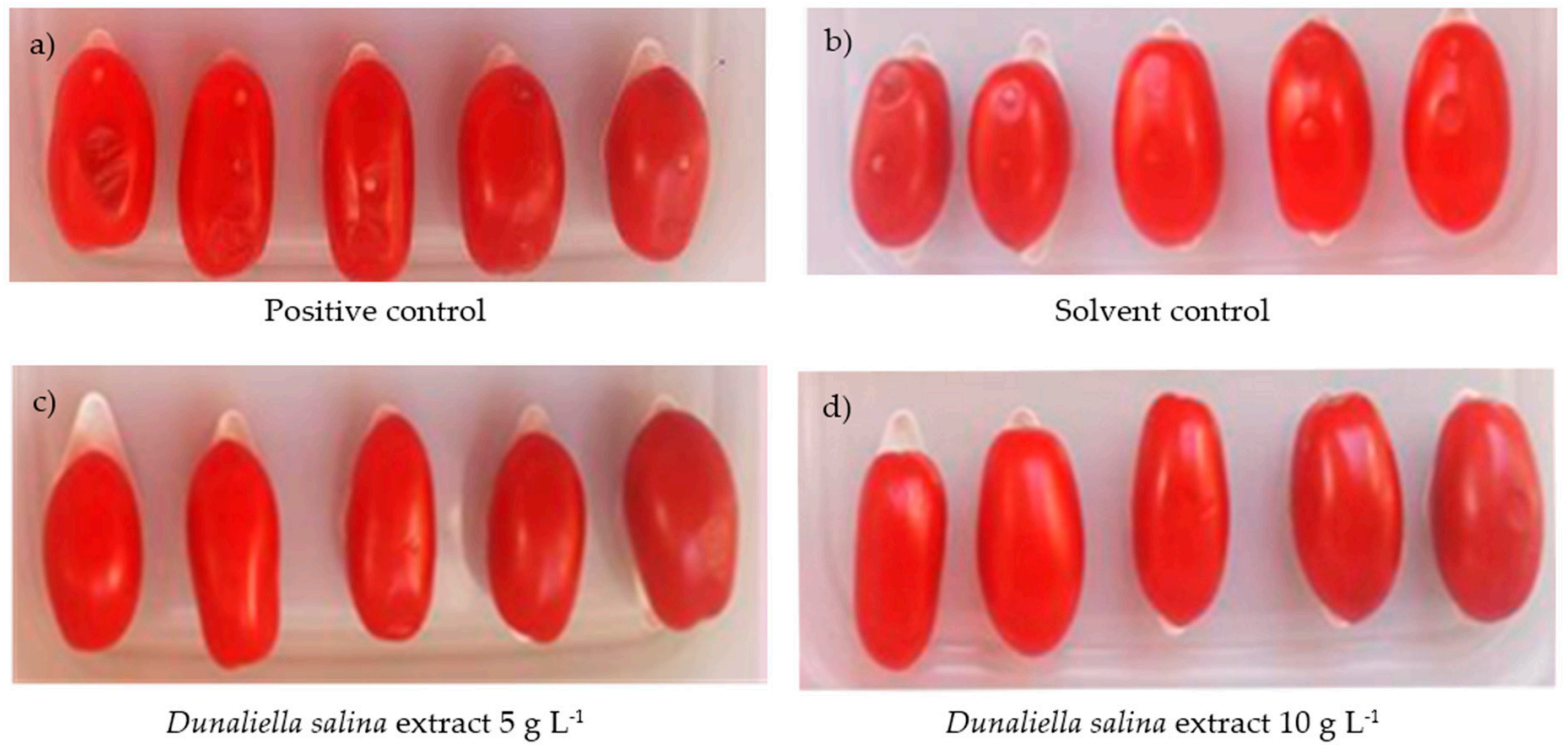
Effect of *D. salina* extracts against *P. carotovorum* subsp. *carotovorum* on tomato fruits: (**a**) positive control, (**b**) solvent control, (**c**) *Dunaliella salina* extract 5 g L^−1^, (**d**) *Dunaliella salina* extract 10 g L^−1^.

Dark-brown spots surrounded by a chlorotic halo appeared 5 days after inoculation with DI and DS of 3.2% and 0.82%, respectively. A final DI and DS of 37.9% and 2.2% were observed. A similar DI and DS not statistically different from those of positive control were recorded. A significant reduction of disease symptoms was observed on leaves of tomato plants treated with *D. salina* hexane at a concentration of 10 mg mL^−1^. In particular, a DI of 0%, 7.2%, and 13.2% after 5, 10, and 15 days were recorded, respectively. At the same time, the DS was 0%, 0.02%, and 0.505%, respectively. No negative (phytotoxic) effects were recorded on tomato plants of the healthy control (Table 5, Figure 2 and Figure 3).

#### 2.3.2. Application of *D. salina* Extracts to Control Bacterial Soft Rot Caused by *P. carotovorum* subsp. carotovorum on Tomatoes and Zucchini Fruits

Antibacterial activity of hexane *D. salina* extracts against *P. carotovorum* subsp. *carotovorum* (PCC) was investigated in vivo. The results in Table 6 show a strong reduction of soft rot symptoms on tomato and zucchini fruits artificially inoculated with the PCC strain and treated with *D. salina* extracts at two different concentrations 10 and 5 mg mL^−1^. In the positive control, tomatoes showed the typical small water-soaked lesion on the inoculation point (Figure 3). Disease incidence was detected equal to 33.4% (Table 6). Tomatoes inoculated with *P. carotovorum* subsp. *carotovorum* and treated with *D. salina* extracts at concentrations of 10 and 5 mg mL^−1^ did not show infected wounds. In the positive control, the number of infected wounds increased, reaching a disease incidence of 80.6%, while a significant antibacterial effect was achieved on tomatoes treated with *D. salina* extracts at concentrations of 10 and 5 mg mL^−1^, measuring a disease incidence of 5.3% and 12.7%, respectively. In solvent control, a disease incidence was recorded equal to 27.7% and 77.9% at 48 and 96 h, respectively. Disease incidence (DI) were not statistically different from those observed on the positive control.

The microalgae extract treatments also resulted in effective control of bacterial soft rot on zucchini inoculated with *P. carotovorum* subsp. *carotovorum*. A significant disease incidence was observed at 96 h at 12.6% and 26.1% in zucchini fruits treated using *D. salina* extracts at the concentrations of 10 and 5 mg mL^−1^, respectively. No significant differences in the percentages of infected wounds were observed between the solvent and the positive controls for all fruits which were completely rotten at the end of the experiments (Table 6). For both trials, the healthy control did not show any symptoms after 96 h from inoculation.

### 2.4. Relationship between β-carotene Concentration and Antibacterial Activity

The results of the values of the inhibition zones for *D. salina* extracts and β-carotene against *P. carotovorum* subsp. *carotovorum*, *P. syringae* pv. *tomato* and *B. subtilis* are shown in Table 7. The β-carotene sample at a concentration of 10 mg mL^−1^ showed high antimicrobial activity against all tested bacteria. Indeed, an inhibition zone of 9.8, 10.5, and 18.1 mm was measured for *P. carotovorum* subsp. *carotovorum*, *P. syringae* pv. *tomato*, and *B. subtilis*, respectively. These value of inhibition zones were not significantly different from those produced by samples of a *D. salina* hexane extract at a concentration of 100 mg mL^−1^.

## 3. Discussion

The control of crop diseases by eco-friendly means and products is among the major priorities for the agricultural and food industries. Pathogenic bacteria are able to infect hosts causing severe diseases to many agricultural crops. The discovery of new natural compounds able to prevent and manage bacterial plant diseases is a crucial step in agriculture research. In this context, in this study, *D. salina* biomass was subjected to solvent extraction by a Dionex ASE 200 extractor with the aim to check extracts’ antibacterial proprieties. The results clearly indicate a significant antibacterial activity with a different inhibition level of each *D. salina* extract. Among the extracts, those obtained using hexane exhibited a better antibacterial effect although the worst extraction yield performance was detected in the same.

In particular, the hexane-based extracts of *D. salina* showed a MIC value of 0.3 mg mL^−1^ for *B. subtilis* and 3.0 mg mL^−1^ for *P. carotovorum* subsp. *carotovorum* and *P. syringae*. The observed difference in microbial sensitivities to the microalgae extracts may be attributed to the fact that cell walls in Gram-positive bacteria consist of a single layer, whereas Gram-negative bacterial cell walls are a multilayered structure bounded by an impermeable outer phospholipid membrane which is an effective barrier against hydrophobic substances [34,35]. Similar MIC values for *D. salina* extracts were recently reported in other independent studies carried out by Jafari et al. [36]. The authors investigated the antibacterial potential of *D. salina* extracts against *Streptococcus mutans* strain PTCC 1683, a Gram-positive bacterium causal agent of dental caries, showing that an inhibitory effect in serial dilution concentrations between 0.75 to 25 mg mL^−1^ started at 6.25 mg mL^−1^. Despite *D. salina* being known for its antimicrobial properties, as reported in the literature [22,37], based on our current knowledge, there are no data about its effectiveness against bacterial plant pathogens.

In addition to the preliminary screening, in vivo trials were performed in order to confirm the promising antibacterial activity shown by the hexane extracts of *D. salina* against two important phytopatogenic bacteria, i.e., *P. syringae* and *P. carotovorum*. The present study showed, for the first time, that treatments with *D. salina* extracts significantly reduced the incidence of bacterial speck spots and bacterial soft rot diseases.

Certainly, in natural conditions without artificial inoculation, it might be hypothesized that a better effectiveness of *D. salina* microalgae extracts to control these two important plant diseases could be achieved.

On the basis of *D. salina* chemical composition it might be presumed that the antibacterial activity was related to β-carotene as a major component. This hypothesis seems to be supported by comparing the antimicrobial activities of *D. salina* extracts with that observed using the chemical standard of β-carotene.

The standard β-carotene showed significant antibacterial activity against *P. syringae*, *P. carotovorum*, and *B. subtilis* and not statistically significant differences were in comparison to the activity of the *D. salina* hexane extracts. Excellent results in antibacterial activity of carotenoids were reported in several independent studies carried out on microalgae and in particular on *D. salina* biomass [20,21,38,39,40,41,42]. In particular, Bhagavathy et al. [43] associated the antimicrobial effects of extracts from green algae, *Chlorococcum humicola*, to major coloring pigment (β-carotene).

Although further efforts are needed to precisely identify the molecules contained in *D. salina* which manifest antimicrobial properties, the presented results highlight that the comparison between antimicrobial activity of *D. salina* extracts with those of the beta-carotene standard would demonstrate that the β-carotene could be the main antibacterial compound in the extract against the tested pathogenic microorganisms.

## 4. Materials and Methods

### 4.1. Extraction and Chemical Characterization of D. salina Microalgae

*Dunaliella salina* biomass was supplied as dry powder with a particle size lower than 50 μm by Algalimento, a Spanish company that cultivate microalgae for commercial applications in food and pharmaceutical industries. The biomass was stored at −20 °C in a plastic bag under vacuum to avoid degradation until characterization and extraction.

Before the chemical extraction, in order to increase the yield, the dry microalgae biomass was added with diatomaceous earth and pretreated mechanically at 500 rpm for 5 min by ball mill of Retsch MM400^®^ (Fisher Scientific, UK) [44,45,46,47]. Subsequently, pressurized fluid extraction (PFE) was carried out by a Dionex ASE^®^ 200 extractor (Salt Lake City, UT, USA). Stainless-steel extraction cells with a volume of 11 mL were filled in consecutive layers with a cellulose filter (20 µm pore size), a 2–3 cm^3^ layer of inert material (diatomaceous earth), 2.0 g pretreated microalgae biomass, and an additional layer of inert material. Extraction cells and collection vials were loaded into the automated carousel and the pre-heating time of the cells was fixed at 5 min. Extracts were collected into 40 mL amber glass vials by flushing the system with 6.6 mL of fresh solvent after each extraction tests. At the end of each extraction, the system was purged for 1 min with nitrogen. Solvents with different polarity were used for the extraction of antimicrobial compounds from *D. salina*. Chloroform:Methanol (1:1), ethanol, and hexane were used as extraction solvents at 50 °C and 100 bar, and the extraction was carried for 2 cycles; each one lasted 10 min. 

The microalgae biomass of *D. salina* and its extracts were characterized in terms of humidity, ash, total dietary fiber (TDFs), carbohydrates, proteins, fatty acids, and carotenoids following standard methods as earlier reported [44]. In particular, the total content of carotenoids was quantified using an Agilent 1290 Infinity II uHPLC equipped with a diode array detector (DAD) and an Agilent Zorbax Eclipse plus C18 column 1.8 µm column. For detection and quantification of individual species, such as lutein, beta-carotene, and astaxanthin, wavelengths of 444 nm, 450 nm, 478 nm, respectively, were used [47]. The chromatographic analyses were ran as indicated in the standard method UNI EN 12823-2 [48] and in the paper by Ruen-ngam et al. [49].

### 4.2. In Vitro Antimicrobial

The phytopathogenic bacterial strains *P. syringae* pv. *tomato* EPS3 strain and *B. subtilis* strain ET-1 were provided by the laboratory of the ENEA Research Centre, Italy, while *P. carotovorum* subsp. *carotovorum* strain DSM30168 was supplied by by DSMZ—German Collection of Microorganisms and Cell Cultures, Germany. The bacterial strains were used for antimicrobial trials in vitro. and The EPS3 was cultured on Medium 54 (glucose 20.0 g L^−1^, yeast extract 10.0 g L^−1^, CaCO_3_ 20.0 g L^−1^, agar 17.0 g L^−1^) at 26 °C, while strains DSM30168 and ET-1 on nutrient agar (NA; Sigma–Aldrich, USA) at 30 °C.

#### 4.2.1. Agar Disc Diffusion Method

The agar disc diffusion method was carried out to evaluate the antimicrobial activity of *D. salina* extracts. The extracts obtained by different solvents were dried at 40 °C by a speed dry vacuum concentrator. The dry weight was determined for each sample after drying and concentration steps. On the basis of their solubility, the extracts were subsequently dissolved in different aliquots of sterile dimethyl-sulfoxide (DMSO).

Briefly, an aliquot of different extracts (10 µL) was loaded on sterile filter paper discs (6 mm in diameter, Whatman no. 03) that were placed on NA and Medium 54 plates inoculated with a bacterial suspension containing 10^5^ cells mL^−1^. For negative, solvent, and positive controls, paper discs containing 10 μL of sterile water, DMSO, and one standard antibiotic (ciprofloxacin) were used, respectively. All plates were incubated at 26 °C for 5–7 days. After the incubation time, the diameter of the inhibition zones around the discs were measured using a digital caliber. For all tested bacteria, three replicates were performed.

#### 4.2.2. Minimum Inhibitory Concentration (MIC)

A broth dilution method was used for determination of the minimum inhibitory concentrations (MICs) of *D. salina* extracts. The dried extracts obtained after evaporating at 40 °C by a speed dry vacuum concentrator were subsequently dissolved in DMSO in order to obtain the following concentration: 16.25, 32.5, 75.0, 150.0, and 400.0 mg mL^−1^. Each sterile tube (1.5 mL) was prepared by dispensing into 193 µL of liquid medium, 2 µL of the different extracts at the two concentrations and 5 µL of the bacteria suspensions containing 1 × 10^5^ cells mL^−1^. For each tube, the final volume was of 200 µL. Liquid medium with DMSO at 1% was used as a negative control. All tubes were incubated at a temperature of 26 °C in a shaker incubator (Thermomixer comfort by Eppendorf). After an incubation period of 2 days, the antibacterial activity was detected by counting the viable cells. For this purpose, 50 µL of cell culture from the different tube were decimally diluted and spread on agar plates. After 48 h of incubation at 26 °C, the number of colonies was counted and compared with the number of viable cells present in the culture broth at the time of inoculation. For each extract, the lowest concentration that clearly prevented the microorganism growth was designated as MIC. Each test was replied three times.

### 4.3. In Vivo Antimicrobial Assay

On the basis of in vitro assay, a volume of *D. salina* hexane extract (100 mL) was dried at 35 °C using a rotary evaporator instrument (Steroglass, Perugia, Italy) and after dry weight was quantified using an analytical balance (KERN & SOHN GmbH, Germany). The dried extracts were dissolved in absolute ethanol to a final concentration of 100 mg mL^−1^ and subsequently diluted with sterilized distilled water up to 10 and 5 g L^−1^.

#### 4.3.1. Application of *D. salina* Extracts to Bacterial Speck Spot caused by *P. syringae*

The effect of *D. salina* extracts was evaluated in vivo on tomato plants. Young plants of *Lycopersicon esculentum* (L.) cv. Tomito F1 (ISI Sementi SPA, Italy), a commercial cultivar without resistance genes to Pst, was used for in vivo trials. The plants were transplanted on plastic pots (28 cm × 40 cm) containing universal substrate (Florarura) and grown in an experimental greenhouse located at ENEA Research Center (Rotondella, Matera) Italy.

The leaves of tomato plants that were 1 month old were gently touched before the treatments and inoculation, in order to produce superficial wounds similar to those caused by transplanting and cultural practices. Preventive treatments were done by spraying *D. salina* extracts (10 mg mL^−1^) through a glass TLC sprayer on the adaxial and abaxial leaf surfaces until their surface became homogeneously wet. Solvent and positive controls have been prepared by spraying plants with an emulsifying solution of ethanol 10% and distilled water, respectively. Healthy control plants were sprayed with physiological water only. After 6 h, bacterial suspension containing 10^7^ cells mL^−1^, previously prepared by centrifuging culture and suspending the cells in physiological water, was spray-inoculated on tomato plants (0.5 mL for each plant). During the experiments day and night temperature were set at 22 °C and 15 °C while, the relative humidity was greater than 80% to support stomata opening. Tomato plants were monitored for 15 days and three replicates were carried out such as each test has been repeated three times. Disease incidence (DI) and disease severity (DS) were evaluated recording the symptoms on 150–200 leaves per treatment. Disease incidence (DI) is the percentage of infected leaves that was determined by the following formula:

DI% = (IL/L) × 100(1)

where IL is the number of infected leaves and L is the number of recorded leaves.

DS was analyzed according to Gullino et al. [50]. In particular, a visual scale from 0 to 5 based on disease symptoms was adopted to score the disease leaves as follows: 0 = no symptom; 1 = up to 5% infected leaf area; 2 = 6% to 10% infected leaf area; 3 = 11% to 25% infected leaf area; 4 = 26% to 50% infected leaf area; 5 = 51% to 100% infected leaf area. It describes the damage caused by the diseases on plants leaves and was calculated by the following formula (2):

DS = ∑ (n° leaves * X_0−5_)/(total of leaves recorded) (2)

with X_0−5_ = (X0 = 0; X1 = 3%; X2 = 8%; X3 = 18%; X4 = 38%; X5 = 75.5%); n leaves: number of leaves for each class.

A randomized complete block design was applied with three replicates thus each test were repeated three times. In each replicate, three treatments (*D. salina* extracts, positive control and solvent control) were considered. For each set one plastic pot with 8 plants was used. The experiments were repeated twice with a total of 96 tomato plants for each experiment (8 plants × 4 treatments × 3 replicates).

#### 4.3.2. Application of *D. salina* Extracts to Control Bacterial Soft Rot caused by *P. carotovorum*

The effectiveness of *D. salina* extracts on bacterial disease caused by *P. carotovorum* subsp. *carotovorum* was investigated with two in vivo trials on fresh tomatoes and zucchini fruits.

Fresh tomato (*Lycopersicon esculentum* cv. Pixel) fruits were selected uniform in size and color, and without wounds. The fruits were washed with tap water and the surface was dipping in 0.1% sodium hypochlorite solution for 10 min, rinsed sterile twice by using distilled water, and dried in ambient air. Four uniform wounds (2 mm deep and 0.5 mm wide) were made in three equidistant points on one side of each fruit. Wounded fruits were immersed for 1 min in *D. salina* extracts formulations at concentration of 5 and 10 g L^−1^. Positive and solvent controls fruits were treated by dipping in distilled water and in 10% ethanol, respectively. Each wound was inoculated with an aliquot (10 µL) of *P. carotovorum* subsp. *carotovorum* (10 ^6^ cells mL^−1^) in physiological water. Wounded fruits that were used as healthy control, were not inoculated and were treated only with physiological water.

Zucchini fruits (*Cucurbita pepo* L. cv Afrodite) wounded making a cut (3 mm deep, 10 mm long) in three equidistant points along a longitudinal line with a sterile scalpel blade. Each wound was impregnated with the two different concentrations of *D. salina* extracts (500 μL). For positive and solvent controls, wounds were impregnated with 500 μL of distilled water and 10% ethanol, respectively. Each wound was inoculated with a 10 µL aliquot containing 10^6^ cells mL^−1^ of *P. carotovorum* subsp. *carotovorum*. Wounded fruits treated only with physiological water were used as healthy control.

Tomatoes and zucchini fruits were placed in a plastic container and incubated at 24 °C and 80–85% relative humidity (RH). Disease incidence was assessed by recording the number of infected wounds at 48 and 96 h after inoculation. In tomatoes, infected and diseased wounds showed rot signs at the inoculation points while, in zucchini a transversal section was made at the inoculation points to detect rot signs. The percentage of DI was calculated using formula (3):

DI% = (IW/W) × 100(3)

where, W is the number of wounds and IW is the number of infected wounds.

A randomized complete design was used for three replicates. For each replicate, four treatments (*D. salina* extracts at concentration of 10 and 5 mg mL^−1^, positive control and solvent control) were considered. Two sets of three replicates: one with 10 tomatoes per replicate and another with 4 zucchini per replicate, were prepared for each treatment (4 treatments × 3 replicates × 10 or 4 fruits for a total of 120 tomatoes and 48 zucchinis for each experiment). The experiments were repeated twice.

### 4.4. β-carotene Concentration and Antibacterial Activity

The antibacterial activity of *D. salina* extracts against *P. syringae*, *P. carotovorum* and *B. subtilis* at concentration of 100 mg mL^−1^ was compared with a β-carotene chemical standard (Sigma Chemical) at concentration of 10, and 5 mg mL^−1^ through the Agar Disc Diffusion method. Briefly, the cells of different bacteria species were removed with sterile loop from 4 days old single colonies and dissolved into 1 mL of sterile distilled water in order to obtain a final concentration of 1 × 10^6^ cells mL^−1^. An aliquot of cell suspension (100 μL) was uniformly spread on the surface of plates (90 mm) containing approximately 20 mL of NA medium. For each bacterial species, a volume of 10 µL of three different extracts of *D. salina* and β-carotene were loaded on paper discs placed on NA plates that were incubated at 25 °C for 7–10 days. After the incubation time, the diameter inhibition zones around the discs were measured using a digital caliber. The assay was repeated three times.

### 4.5. Statistical Analyses

The effects of *D. salina* extracts (independent variables) on the two bacterial diseases (dependent variables) were examined using Statistical Analysis System software (SAS Institute Inc., Cary, NC, USA). Prior to analysis, the data were verified for homogeneity of variance and for normal distribution. In each in vivo trial, the data of the two repeated experiments were pooled and analyzed as a completely randomized design. Means were separated by Tukey’s HSD test when the analysis of variance showed statistical significance (α = 0.05).

## 5. Conclusions

In this study, the extracts obtained from the microalgae *D. salina* by using pressurized fluid extraction technology and using solvents with different polarities show antibacterial activity. The best performances in vitro have been found for hexane extracts. On young tomato plants, *D. salina* extracts) reduced up to 65.7% and 77.0% in incidence and severity, respectively, of bacterial speck spot disease. Similarly, it was observed a strong reduction of soft rot symptoms on tomato and zucchini fruits artificially inoculated and treated with two different concentrations of extracts. In particular, treated tomatoes and zucchini showed a disease incidence of 5.3% and 12.6%, respectively, while at same time on the corresponding positive controls, the disease incidence achieved 80.6% for tomato and 100% for zucchini.

## Figures and Tables

**Table 1:** Chemical composition of *Dunaliella salina* and fatty acids composition.

Chemical–Physical Features	
Humidity *	6.63 ± 0.25
Ash ^#^	48.74 ± 2.50
Proteins ^#^	10.03 ± 0.57
Carbohydrates ^#^	25.31 ± 1.55
Lipids ^#^	3.49 ± 0.10
Total Dietary Fibers ^#^	8.97 ± 0.50
Carotenoids ^#^	3.46 ± 0.15
Fatty acids methyl esters composition (mg 100 g^−1^ on dry basis)	
**SFAs ^§^**	
Tridecanoic acid	<Ldl
Palmitic acid	965.00 ± 1.15
Pentadecanoic acid	<Ldl
Heptadecanoic acid	<Ldl
Stearic acid	567.68 ± 0.56
Arachidic acid	<Ldl
∑ SFAs	1532.68 ± 1.70
**MUFAs ^§^**	
Palmitoleic acid	<Ldl
cis-9-Octadecenoic acid (oleic acid)	567.56 ± 1.29
Myristoleic acid	<Ldl
Nervonic acid	<Ldl
Erucic acid	<Ldl
∑ MUFAs	567.56 ± 1.29
**PUFAs ^§^**	
cis-8,11,14-Eicosatrienoic acid	<Ldl
Linoelaidic acid	<Ldl
Linoleic acid	519.75 ± 0.63
γ-Linolenic acid	536.22 ± 0.12
Arachidonic acid	<Ldl
cis-5,8,11,14,17-Eicosapentaenoic acid	<Ldl
∑ PUFAs	1055.97 ± 0.75

* %*w*/*w* on wet sample; # %*w*/*w* on dry basis; § SFAs: saturated fatty acids; MUFAs: monounsaturated fatty acids; and PUFAs: polyunsaturated fatty acids. All data are the mean value ± standard deviation. Ldl = lower detection limit.

**Table 2:** Chemical characterization of *D. salina* extracts using solvents with different polarity.

Compounds	Chloroform:Methanol (1:1)	Ethanol	Hexane
Ash	30.3%	32.5%	36.2%
Protein	26.4%	24.8%	14.1%
Carbohydrates	3.0%	2.6%	1.4%
TDF	9.1%	8.5%	5.6%
Carotenoids	16.4%	12.8%	36.6%
of which:			
Beta-carotene	85.0%	90.0%	98.0%
Lutein	15.0%	10.0%	2.0%
Lipids	14.8%	18.8%	6.1%
of which FAMEs:	90.1%	85.3%	90.0%
FAMEs composition:			
SFAs	30.1%	35.0%	32.0%
MUFAs	60.1%	56.7%	65.9%
PUFAs	9.8%	8.3%	2.1%

**Table 3:** Antibacterial activity of *D. salina* extracts by disc diffusion method.

Samples	Concentration (mg mL^−1^)	Inhibition Zone (mm)		
		*P. carotovorum* subsp. *carotovorum* DSM30168	*P. syringae* pv. *tomato* EPS3	*B. subtilis* ET-1
Chloroform:Methanol extract	350.0	10.0 ± 0.1	8.0 ± 0.1	13.0 ± 0.1
Ethanol extract	214.0	11.0 ± 0.1	9.0 ± 0.1	21.0 ± 0.2
Hexane extract	97.0	9.0 ± 0.1	12.0 ± 0.1	20.0 ± 0.2
Ciprofloxacin	0.15	20.0 ± 0.2	24.0 ± 0.2	32.0 ± 0.3

**Table 4:** Minimum inhibitory concentration (MIC) of *D. salina* extract against *P. carotovorum*, *P. syringae*, and *B. subtilis.*

	MIC Value (mg mL^−1^)		
	*P. carotovorum*	*P. syringae*	*B. subtilis*
Chloroform:Methanol	>3.0	>3.0	3.0
Ethanol	>3.0	>3.0	3.0
Hexane	3.0	3.0	0.3

**Table 5:** Disease incidence (DI) and disease severity (DS) of bacterial specks on inoculated tomato plants at 5, 10, and 15 days post-inoculation.

Treatments	5		10		15	
	DI (%)	DS (%)	DI (%)	DS (%)	DI (%)	DS (%)
Positive Control	3.2 ± 0.1 ^b*^	0.82 ± 0.3 ^b^	25.2 ± 0.1 ^b^	1.8 ± 0.3 ^b^	37.9 ± 0.2 ^b^	2.2 ± 0.1 ^b^
Solvent Control	3.5 ± 0.1 ^b^	0.93 ± 0.2 ^b^	24.2 ± 0.1 ^b^	1.85 ± 0.1 ^b^	36.7 ± 0.9 ^b^	2.9 ± 0.2 ^b^
Hexane extract	0.0 ^a^	0.0 ^a^	7.2 ± 0.5 ^a^	0.02 ± 0.1 ^a^	13.2 ± 0.4 ^a^	0.505 ± 0.1 ^a^

* Data are shown as the means ± standard deviation. Within each column, mean values followed by the same letter (a, b) are not significantly different according to Tukey’s test (α = 0.05).

**Table 6:** Disease incidence (DI) on tomato and zucchini fruits after 48 and 96 h from inoculation.

Treatments	Disease Incidence (%)			
	Tomatoes Fruits		Zucchini Fruits	
	Incubation Time (hours)			
	48	96	48	96
Positive control	33.4 ± 0.32 ^a*^	80.6 ± 0.56 ^b^	90.4 ± 0.33 ^a^	100.0 ^b^
Solvent control	27.7 ± 0.32 ^a^	77.9 ± 0.43 ^b^	86.2 ± 0.23 ^a^	100.0 ^b^
Extract 10 mg mL^−1^	0.0 c ± 0.0	5.3 ± 0.23 ^c^	0.0 c ± 0.0	12.6 ± 0.15 ^c^
Extract 5 mg mL^−1^	0.0 c ± 0.0	12.7 ± 0.12 ^a^	0.0 c ± 0.0	26.1 ± 0.22 ^a^

* Data are shown as the means ± standard deviation. Within each column, mean values followed by the same letter (a, b, c) are not significantly different according to Tukey’s test (α = 0.05).

**Table 7:** Antibacterial activity of *D. salina* extracts at a concentration of 100 mg mL^−1^ and β-carotene (chemical standard) at concentrations of 10, 5, and 3 mg mL^−1.^

			Inhibition Zone (mm)		
Sample	Sample Concentration (mg mL^−1^)	β-carotene Concentration (mg mL^−1^)	*P. carotovorum* subsp. *carotovorum*	*P. syringae* pv.	*B. subtilis*
β-carotene SD		10	9.8 ± 0.5 ^a*^	10.5 ± 0.4 ^a^	18.1 ± 0.2 ^a^
β-carotene SD		5	5.1 ± 0.3 ^b^	8.4 ± 0.3 ^b^	9.3 ± 0.2 ^c^
Chloroform:Methanol extract	100	13.9	6.2 ± 0.2 ^b^	7.7 ± 0.3 ^b^	9.1 ± 0.6 ^c^
Hexane extract	100	35.9	10.5 ± 0.8 ^a^	11.2 ±0.7 ^a^	19.7 ± 0.2 ^a^
Ethanol extract	100	11.5	7.2 ± 0.2 ^b^	6.4 ± 0.4 ^c^	14.2 ± 0.8 ^b^

* Data are shown as the means ± standard error. In column, the means followed by a different letter (a, b, c) are significantly different according to the Tukey’s HSD test (α = 0.05).
